# Striving toward quality metrics for pediatric stroke: time from door to diagnosis

**DOI:** 10.3389/fstro.2025.1718355

**Published:** 2026-01-07

**Authors:** Rachel Pearson, Nancy K. Hills, Kellie Bacon, Shelby K. Shelton, Rowena Roque, Tatiana Moreno, Maria Kuchherzki, Carl Schultz, Theodore W. Heyming, Christine K. Fox, Heather J. Fullerton

**Affiliations:** 1 Rady Children's Health, Orange County, Orange, CA, United States; 2 UC Irvine School of Medicine, University of California, Irvine, Irvine, CA, United States; 3 Departments of Neurology and Pediatrics, University of California, San Francisco, San Francisco, CA, United States; 4 Orange County Healthcare Agency, Santa Ana, CA, United States

**Keywords:** pediatric stroke, prehospital/EMS, stroke care pathways, acute stroke care, stroke diagnosis

## Abstract

**Background/Objective:**

Most pediatric stroke survivors suffer long-term impairments. To minimize injury, it is essential to quickly restore perfusion to viable brain tissue. Minimizing the time to stroke diagnosis requires recognition of a possible stroke by prehospital and emergency healthcare personnel, and rapid neuroimaging. While CT suffices for diagnosing hemorrhagic stroke, MRI is necessary to diagnose acute ischemic stroke (IS), contributing to significant diagnostic delays and potentially missed opportunities for intervention.

**Methods:**

We conducted a retrospective study of children 1–14 years old with acute neurological symptoms presenting by Emergency Medical Services (EMS) to the study institution from 1/2019–6/2023. We described patient characteristics and neuroimaging studies, then evaluated predictors of MRI acquisition and actionable findings, including stroke. To assess the generalizability of these data we analyzed a secondary retrospective cohort of all children admitted during this period with out-of-hospital strokes regardless of presentation modality [EMS, emergency department (ED) walk-in, and transfer].

**Results:**

Among 3,888 pediatric patients with acute neurological symptoms presenting via EMS, 695 (17.9%) had neuroimaging: CT only in 570 patients (14.7%); CT and MRI in 125 (3.2%). Median (IQR) times from EMS activation to neuroimaging were 2.29 (1.56, 3.21) hours for CT and 26.8 (16.3, 43.8) hours for MRI. An EMS primary impression of “stroke” was rare (*n* = 13) but strongly predictive of imaging acquisition: all had CT and 11 had MRI. Thirty-one of the 125 patients with MRI had actionable MRIs, including nine acute strokes. During the study period another 14 stroke patients presented as ED walk-ins. Median time from ED arrival to CT was 0.92 (0.47, 1.08) hours for EMS patients with hemorrhagic stroke and 5.69 (1.50, 9.76) hours for walk-ins; for MRI, median time was 4.15 (3.00, 5.31) hours for EMS patients with ischemic stroke and 10.2 (1.99, 36.3) hours for walk-ins.

**Conclusion:**

Among children with acute neurological symptoms selected for neuroimaging, CT was the most common modality while MRIs were performed with a substantial time delay. While EMS providers rarely suspected stroke, their diagnosis impacted imaging decisions in the ED, suggesting a need to raise awareness among prehospital providers. To measure quality improvement in pediatric stroke, new pediatric-specific metrics like “door to diagnosis” time, should be further explored.

## Introduction

Adult stroke care has been transformed through quality improvement research and the use of common metrics that are tracked to ensure best practices, as recommended by the American Heart Association (AHA) and the American Stroke Association (ASA)'s “Get with the Guidelines” initiative. These quality metrics include the door-to-needle time within 60 min and door-to-puncture time within 90 min (Ormseth et al., 2017). Such metrics have standardized and elevated the quality of adult stroke care across primary stroke centers and comprehensive stroke centers throughout the nation (Reeves et al., 2024).

The metrics tracked for adult stroke, however, are likely not appropriate for the pediatric population, for several reasons. First, common etiologies of pediatric stroke differ from those in adults, with a greater proportion of pediatric stroke being hemorrhagic rather than ischemic, and, within pediatric arterial ischemic stroke, a greater proportion being secondary to arteriopathy (Ferriero et al., 2019; Wintermark et al., 2014). In addition, the frequency of stroke mimics is much higher in children, so the pre-test probability of stroke in a child is much lower than in an adult, even if a child presents with focal neurological deficits (Mackay et al., 2016; Jordan and Hillis, 2011). For this reason, and due to the lack of safety and efficacy data on thrombolytics in the pediatric population, most pediatric stroke experts recommend that stroke be confirmed on magnetic resonance imaging (MRI) prior to administration of thrombolytic therapy. In contrast, the standard of care for adults is to consider thrombolytic therapy in patients that have a clinical presentation consistent with stroke and no bleed on CT or other contraindications (Jordan and Hillis, 2011; Harrar et al., 2022; Fox, 2023; Rivkin et al., 2015). Thus MRI is of primary diagnostic importance in pediatric patients, while CT is of primary diagnostic importance in adult patients.

These factors, in addition to a general lack of awareness of pediatric stroke amongst lay people and healthcare professionals, have contributed to marked delays in the diagnosis of pediatric stroke (Srinivasan et al., 2009). The median time from symptom onset to diagnosis of childhood arterial ischemic stroke is approximately 24 hours (Mallick et al., 2015; Rafay et al., 2009; Hori et al., 2019). This diagnostic delay has led many pediatric hospitals to develop and implement pediatric stroke care pathways as a step toward improved care for children with stroke (Harrar et al., 2022). Now that such pathways are widely used, we must define pediatric-specific metrics that can help us assess the effectiveness of such pathways and identify opportunities for process improvement. Pediatric stroke neurologists have proposed use of time to diagnostic imaging as a key metric instead of time to intervention (door-to-needle times in adults) (DeLaroche et al., 2016; Fearn et al., 2025; Mackay et al., 2017). To explore the feasibility and utility of time to diagnosis as a metric for pediatric stroke, we first sought to better understand imaging practices for children presenting to the hospital via Emergency Medical Service (EMS, i.e. 9-1-1) for acute neurological symptoms.

## Materials and methods

### Primary cohort identification and validation

We performed a retrospective cohort study of all children who presented by ambulance with acute neurological symptoms to a level 1 Pediatric Trauma center located in Orange County, California, during the period from January 2019 to June 2023. Orange County has a population of 3.1 million residents; it is a mostly urban county with 34 incorporated cities. Orange County is an ideal setting for studying EMS practices because it is managed by a single Local Emergency Medical Services Agency, and thus all ambulance care is covered under the same protocols (while other U.S. counties often have multiple services with variable practices). The study institution is the largest of only two Comprehensive Children's Emergency Receiving Centers (CCERC) in Orange County, and the only Pediatric Level I Trauma Center; it receives the majority of high acuity pediatric patients in Orange County who are transported by ambulance for emergency care. The study institution has 24/7 MRI capability, however, during after-hours (Monday through Friday from 8 p.m.−6 a.m., weekends from 6 p.m.−7 a.m., and holidays) an MR technician must be called in to perform the MRI. MRI technicians must arrive at the hospital within 60 minutes of being called in for a study.

Children were identified through electronic queries of EMS records utilizing EMS primary symptom codes, primary impression codes, narrative terms in the EMS report, and dispatch chief complaints that suggested an acute neurological symptom (see Supplementary material). To capture all potential stroke patients, codes for symptoms and conditions that may be a missed stroke diagnosis (i.e., intoxication, head pain due to injury, etc.) were also included. The “primary symptom code” and “primary impression code” are standardized codes entered by EMS personnel after they assess a patient to summarize the patient's symptoms and suspected diagnosis, respectively.

We first identified all patients 1–14 years of age with an EMS activation for acute neurological symptoms who were transported to the study institution's emergency department (ED) by EMS during the period from January 2019 to June 2023. Children over 14 years of age were excluded because they are treated as adults per local EMS guidelines. Children under 1 year of age were excluded because this age group is typically not considered eligible for hyperacute ischemic stroke intervention. We accessed the electronic medical records (EMR) and excluded those with a diagnostic code for traumatic hemorrhage or epilepsy. The study team then reviewed the individual EMS records of the patients selected for the cohort to validate that they met inclusion and exclusion criteria.

### Collection of clinical data

After the final cohort was validated, trained clinical research coordinators performed chart abstraction using the standard hospital EMR and the EMS electronic Patient Care Report (ePCR) which is the electronic medical record from the prehospital provider that is uploaded into the patient's hospital record. They abstracted data on the patient's presentation to EMS including primary symptom code and primary impression code, time of arrival to the ED, transport type, ED variables such as demographics (age, sex, race, ethnicity), chief complaint, Emergency Severity Index, patient past medical history, head imaging types and times, disposition from the ED, and final visit diagnosis for entry into the institution's version of Research Electronic Data Capture (REDCap).

#### Neuroimaging, “actionable findings,” and stroke diagnosis

We defined “neuroimaging” as either CT or MRI of the head acquired during the ED visit or the subsequent hospital admission (if admitted). For patients presenting via EMS, we calculated the time from EMS activation to the acquisition of imaging. A single pediatric neurologist (R.P.) reviewed all MRI reports. If an abnormality was noted, she performed chart review to determine whether or not the finding was “actionable,” defined as a finding that led to a change in medical management. Stroke was defined as a neuroimaging finding of an infarct or hemorrhage (intraparenchymal, intraventricular, or subarachnoid) with acute neurological symptoms that corresponded to the imaging finding. If an infarct or hemorrhage was noted on the imaging report, the same pediatric neurologist performed chart review to classify whether the patient met diagnostic criteria for stroke. For patients with stroke, we calculated the time from ED arrival to diagnostic imaging (“door to diagnosis”).

#### Secondary cohort: children with out-of-hospital stroke

Lastly, to understand the larger context of imaging practices for all children with out-of-hospital acute stroke (regardless of how they presented to medical care), we identified all patients 1–14 years of age who were admitted to the study institution with an acute stroke diagnosis during the study period. We identified this cohort through an electronic search of ED and hospital discharge diagnoses for ICD-10 codes consistent with stroke (see Supplementary material). The same pediatric neurologist performed chart-review of each potential case to confirm the patient had an acute, out-of-hospital stroke, using the same stroke definition as above. She cross-referenced with the previous EMS cohort to ensure there were no duplicate cases. Patients whose strokes occurred in hospital or who had a traumatic hemorrhage or epilepsy diagnosis were excluded. We excluded patients whose stroke was primarily due to trauma (for example, traumatic cerebral hemorrhage in context of a motor vehicle accident). Patients with known history of epilepsy were excluded, as these patients often present to the ED with breakthrough seizures, and providers may approach imaging differently when there is a known history of epilepsy/seizure.

We classified the modality of presentation via chart review: arrival via EMS; arrival as ED “walk-in” (i.e., transported by family/caregiver); or transfer from another facility. We abstracted data on the timing and/or type of neuroimaging. We also measured door to diagnosis time in this cohort to compare those presenting via EMS vs. walk-ins to the ED.

### Statistical analysis

Summary statistics were used to describe the cohort of children with EMS activation for acute neurological symptoms. To identify predictors of neuroimaging, we stratified the cohort by acquisition of neuroimaging (yes/no) and then by type of imaging (CT/MRI). Within the group of children who received MRI (with or without CT), imaging times were summarized using median (IQR) and compared between EMS and non-EMS groups using Wilcoxon rank-sum tests. In the secondary cohort of children with out-of-hospital strokes, we analyzed how modality of presentation (EMS vs. walk-in) affected neuroimaging acquisition.

To compare dichotomous variables, we used chi-square tests or Fisher's exact test, when appropriate. We used Wilcoxon rank-sum tests for categorical variables. An alpha of ≤ 0.05 was used to define statistical significance. All analyses were conducted using Stata v.17.0 (College Station, Texas).

## Results

**Figure 1:**
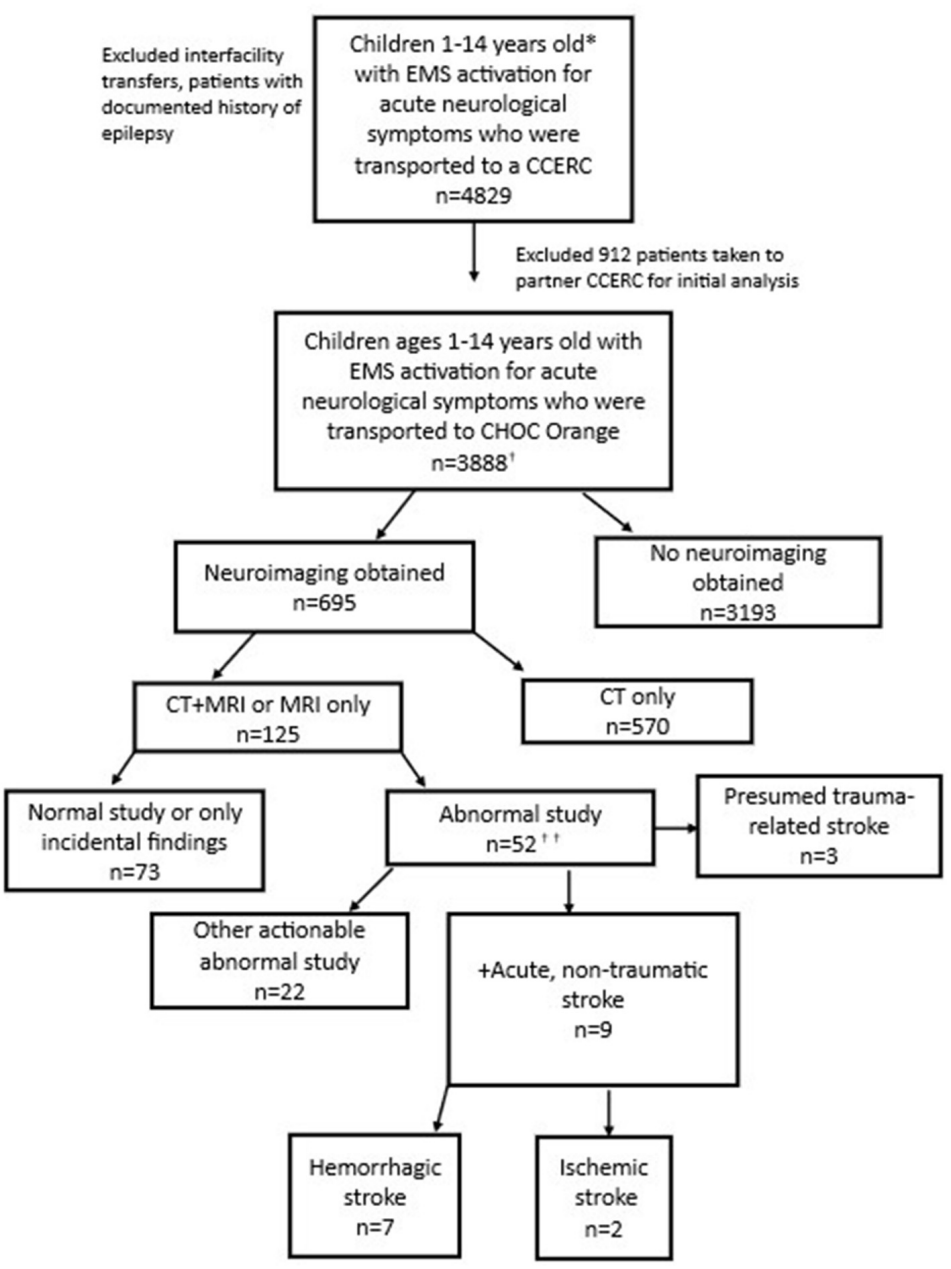
Patients with EMS activation for acute neurological symptoms (Consort diagram). *The county EMS system defines a pediatric patient as under 15 years old. ^†^Includes 2 additional stroke patients brought in by ambulance who were identified via ICD-10 code search but not captured through initial EMS data query. ^††^See Figure 2 for additional details.

Among the primary cohort of 3,888 participants, 695 (17.8%) received neuroimaging: 570 (14.6%) received CT scan only; 125 (3.2%) received CT and MRI (Figure 1). None had MRI only. Median (interquartile range [IQR]) times from EMS activation to CT and MRI were 2.29 (1.56, 3.21) hours and 26.8 (16.3, 43.8) hours, respectively.

### Primary cohort: predictors of neuroimaging

Factors associated with the acquisition of neuroimaging included age 5 years or older (*p* < 0.001) and known medical conditions associated with increased stroke risk (*p* < 0.001), particularly congenital heart disease and trauma (Table 1). All 13 children with a “stroke” EMS primary impression code had neuroimaging. Children with Hispanic ethnicity were less likely to have neuroimaging compared to children of other races or ethnicities (*p* < 0.001), despite no difference in primary language or any other demographic factors.

**Table 1:** Imaging in children with EMS activation for acute neurological symptoms.

**Characteristics**	**Total (*N* = 3,888)**	**No neuroimaging (*****N*** = **3,193)**		**Any imaging (*****N*** = **695)**		***p*-value**
	***N*** **(%)**	***N***	**(%)** ^‡^	***N***	**(%)** ^‡^	
Age in years, Median (IQR)	4.5	4.1	(2.0, 10.5)	6.7	(3.2, 11.9)	0.36^†^
**Age, categorized**						<0.001
<5 years	2,075	1,792	(86.4)	283	(13.6)	
≥5 years	1,813	1,401	(77.3)	412	(22.7)	
**Gender**						0.036
Female	1,831	1,515	(82.7)	316	(17.3)	
Male	2,056	1,678	(81.6)	378	(18.4)	
**Race**						0.15
White	2,128	1,754	(82.4)	374	(17.6)	
Black or African American	131	98	(74.8)	33	(25.2)	
Asian	497	413	(83.1)	84	(16.9)	
Other	1,132	928	(82.0)	204	(18.0)	
**Ethnicity**						<0.001
Hispanic or Latino	2,099	1,774	(84.5)	325	(15.5)	
Multiple	104	85	(81.7)	19	(18.3)	
Not Hispanic or Latino	1,685	1,334	(79.2)	351	(20.8)	
**Past medical history**						
CHD	23	12	(52.2)	11	(47.8)	<0.001
Trauma	24	11	(45.8)	13	(54.2)	<0.001
Sickle cell disease	1	1	(100.0)	0		1.00^**^
Trisomy21	5	2	(40.0)	3	(60.0)	0.04^**^
Malignancy	17	4	(23.5)	13	(76.5)	<0.001^**^
AI disease	10	7	(70.0)	3	(30.0)	0.40^**^
**Primary impression code**						
Altered level of consciousness	397	244	(61.5)	153	(38.5)	<0.001
Anxiety reaction or anxiety	306	292	(95.4)	14	(4.6)	<0.001
Drug overdose/poisoning/alcohol intoxication	168	156	(92.9)	12	(7.1)	<0.001
Seizure or post-ictal	2,787	2,335	(83.8)	452	(16.2)	<0.001
Stroke (CVA/TIA)	13	0		13	(100.0)	<0.001^**^
Other^*^	142	111	(78.2)	31	(21.8)	0.45
**Primary symptom code**					
Psychiatric symptoms	338	306	(90.5)	32	(9.5)	<0.001
Altered mental status and confusion	721	517	(71.7)	204	(28.3)	<0.001
Focal neurological deficits	102	74	(72.5)	28	(27.5)	0.02
Seizure	2,062	1,725	(83.7)	337	(16.3)	0.008
Dizziness	60	56	(93.3)	4	(6.7)	0.02^**^
Drowsiness	42	36	(85.7)	6	(14.3)	0.54
Head Pain (due to injury)	34	16	(47.1)	18	(52.9)	<0.001
Headache	54	48	(88.9)	6	(11.1)	0.13
Systemic symptoms	217	199	(91.7)	18	(8.3)	<0.001
Other (includes no complaint, not recorded, n/a)	103	91	(88.3)	12	(11.7)	0.095

^*^All p-values calculated using Fisher's exact test unless otherwise indicated.

^**^p-value calculated using Chi-squaretest.

^†^p-value calculated using Wilcoxon rank sumtest.

^‡^All percentages are row percentages.

### Primary cohort: predictors of MRI acquisition and abnormal MRI

In the subgroup of children who received any neuroimaging, children who had history of malignancy or an EMS primary impression code of “stroke” were more likely to undergo MRI (*p* < 0.001; Table 2). Children with EMS primary impression codes of “focal neurological deficit” (*p* = 0.01), “altered level of consciousness” (*p* = 0.001), or “anxiety” (*p* < 0.001) were less likely to undergo MRI.

**Table 2:** Imaging in children with EMS activation for acute neurological symptoms: CT only vs. CT/MRI.

**Characteristics**	**Total (*N* = 695)**	**CT only (*****N*** = **570)**		**CT** + **MRI (*****N*** = **125)**		*p*-value^*^
		***N***	**(%)** ^‡^	***N***	**(%)** ^‡^	
Age, median (IQR)	695	6.8	(3.2, 12.0)	5.9	(3.1, 11.0)	0.18^†^
**Age, categorized**						0.53^**^
<5 years	283	229	(80.9)	54	(19.1)	
≥5 years	412	341	(82.8)	71	(17.2)	
**Gender**						0.68^**^
Female	316	257	(81.3)	59	(18.7)	
Male	378	312	(82.5)	66	(17.5)	
**Race**						0.43^**^
White	374	314	(84.0)	60	(16.0)	
Black or African American	33	28	(84.8)	5	(15.2)	
Asian	84	66	(78.6)	18	(21.4)	
Other	204	162	(79.4)	42	(20.6)	
**Ethnicity**						0.91
Hispanic or Latino	325	268	(82.5)	57	(17.5)	
Multiple	19	15	(78.9)	4	(21.1)	
Not Hispanic or Latino	351	287	(81.8)	64	(18.2)	
**Past medical history**						
CHD	11	9	(81.8)	2	(18.2)	0.99
Trauma	13	9	(69.2)	4	(30.8)	0.23
Sickle cell disease	0	0		0		
Trisomy21	3	3	(100.0)	0		0.42
Malignancy	13	6	(46.2)	7	(53.8)	0.001^**^
AI disease	3	2	(66.7)	1	(33.3)	0.49
**Primary impression code**						
Altered level of consciousness	153	127	(83.0)	26	(17.0)	0.001^**^
Anxiety reaction or anxiety	14	12	(85.7)	2	(14.3)	0.001
Drug overdose/poisoning/alcohol intoxication	12	12	(100.0)	0		0.14
Seizure or post-ictal	452	376	(83.2)	76	(16.8)	0.27^**^
Stroke (CVA/TIA)	13	2	(15.4)	11	(84.6)	<0.001
Other^*^	31	25	(80.6)	6	(19.4)	0.84^**^
**Primary symptom code**						
Psychiatric symptoms	32	28	(87.5)	4	(12.5)	0.29
Altered mental status and confusion	204	165	(80.9)	39	(19.1)	0.62^**^
Focal neurological deficits	28	18	(64.3)	10	(35.7)	0.01^**^
Seizure	337	277	(82.2)	60	(17.8)	0.90^**^
Dizziness	4	4	(100.0)	0		1.00
Drowsiness	6	5	(83.3)	1	(16.7)	1.00
Head Pain (due to injury)	18	17	(94.4)	1	(5.6)	0.22
Headache	16	12	(75.0)	4	(25.0)	0.51
Systemic symptoms	18	16	(88.9)	2	(11.1)	0.75
Other (includes no complaint, not recorded, n/a)	12	11	(91.7)	1	(8.3)	0.70

^*^All p-values calculated using Fisher's exact test unless otherwise indicated.

^**^p-value calculated using Chi-square test.

^†^p-value calculated using Wilcoxon rank sum test.

^‡^All percentages are row percentages.

**Figure 2:**
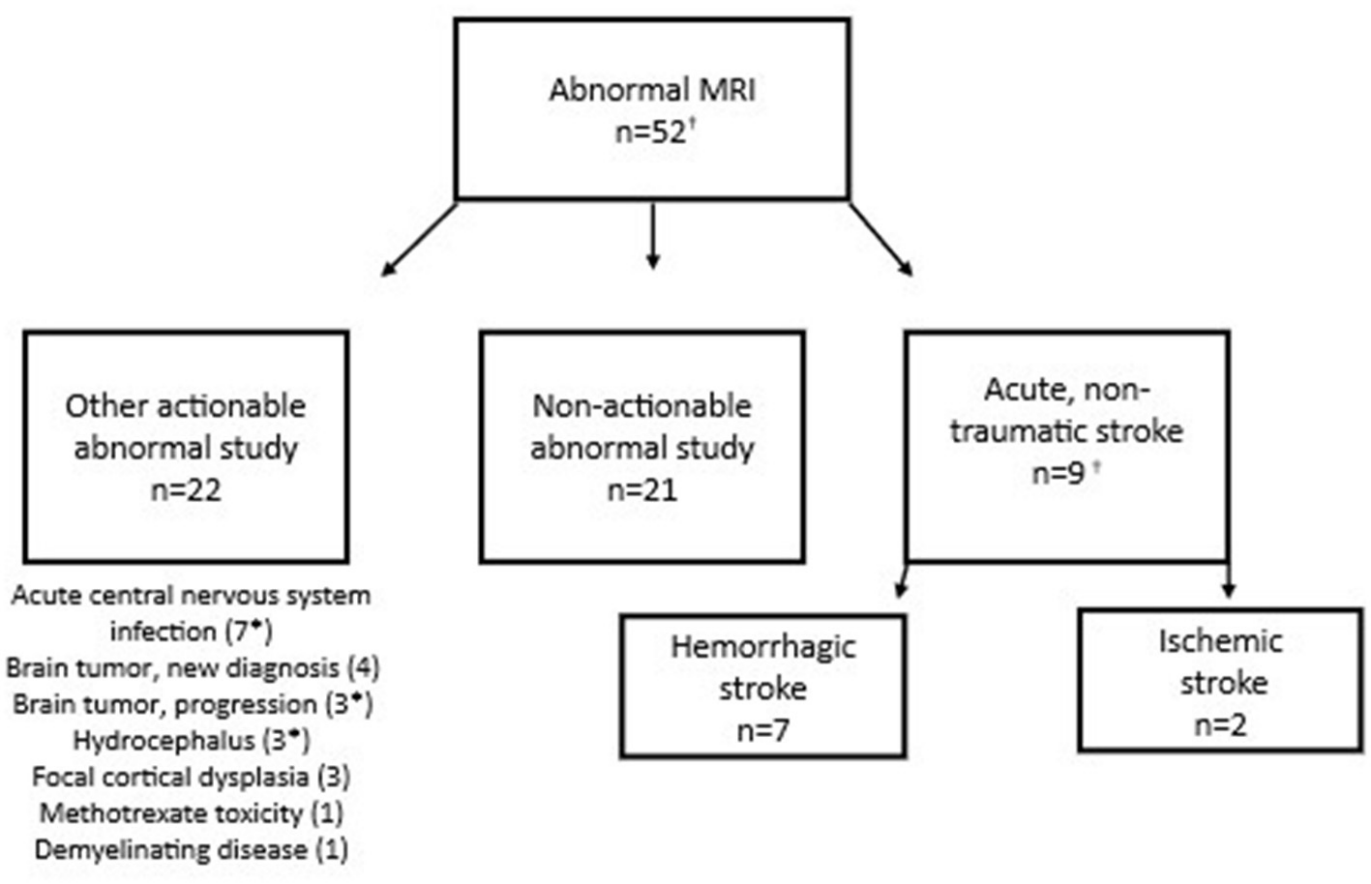
Findings in children with acute neurological symptoms and abnormal MRI. *Subject had more than 1 actionable MRI finding. ^†^Includes 2 additional stroke patients brought in by ambulance who were identified via ICD-10 code search but not captured through initial EMS data query.

Of 125 patients who underwent MRI, 31 (24.8%) had *actionable* abnormal findings (Figure 2). The most common actionable finding was stroke (*n* = 9), followed by acute central nervous system infection (*n* = 7) and brain tumor (*n* = 4). EMS primary symptom and impression codes that were most consistently associated with actionable MRI findings were headache; all 4 patients with headache selected for MRI had an actionable finding (Table 3).

**Table 3:** Primary impression and symptom codes stratified by actionable vs. non-actionable MRI.

**Code**	**Total MRI (*****N*** = **125)**		**Actionable MRI (*****N*** = **31)**		**Non-actionable MRI (*****N*** = **94)**	
	***N***	**(%)**	***N***	**(%)**	***N***	**(%)**
**Primary impression code**						
Other^*^	10	(8.0)	7	(70.0)	3	(30.0)
Stroke (CVA/TIA)	11	(8.8)	3	(27.3)	6	(54.5)
Altered level of consciousness	26	(20.8)	6	(23.1)	20	(76.9)
Seizure or post-ictal	76	(60.8)	15	(19.7)	61	(80.3)
Anxiety reaction or anxiety	2	(1.6)	0		2	(100.0)
Drug overdose/poisoning/alcohol intoxication	0		0			
**Primary symptom code**						
Headache	4	(3.2)	4	(100.0)	0	
Focal neurological deficits	4	(3.2)	2	(50.0)	2	(50.0)
Drowsiness	2	(1.6)	1	(50.0)	2	(100.0)
Other^**^	12	(9.6)	3	(25.0)	9	(75.0)
Altered mental status and confusion	39	(31.2)	9	(23.1)	30	(76.9)
Seizure	60	(48.0)	12	(20.0)	48	(80.0)
Psychiatric symptoms	4	(3.2)	0		4	(100.0)

^*^Includes cardiac arrest (n = 1), fever/cough (n = 1), general weakness (n = 1), headache (n = 3), no apparent illness (n = 1), respiratory arrest (n = 1), syncope (n = 1), other (n =1).

^**^Includes feeding difficulties (n = 1), fever (n = 1), head pain due to injury (n = 1), no complaint (n = 1), pain chest/cardiac (n = 1), respiratory arrest (n = 1), skin numbness (n = 1), weakness (n =5).

### Secondary cohort: presentation modality and imaging characteristics in children with acute stroke

**Figure 3:**
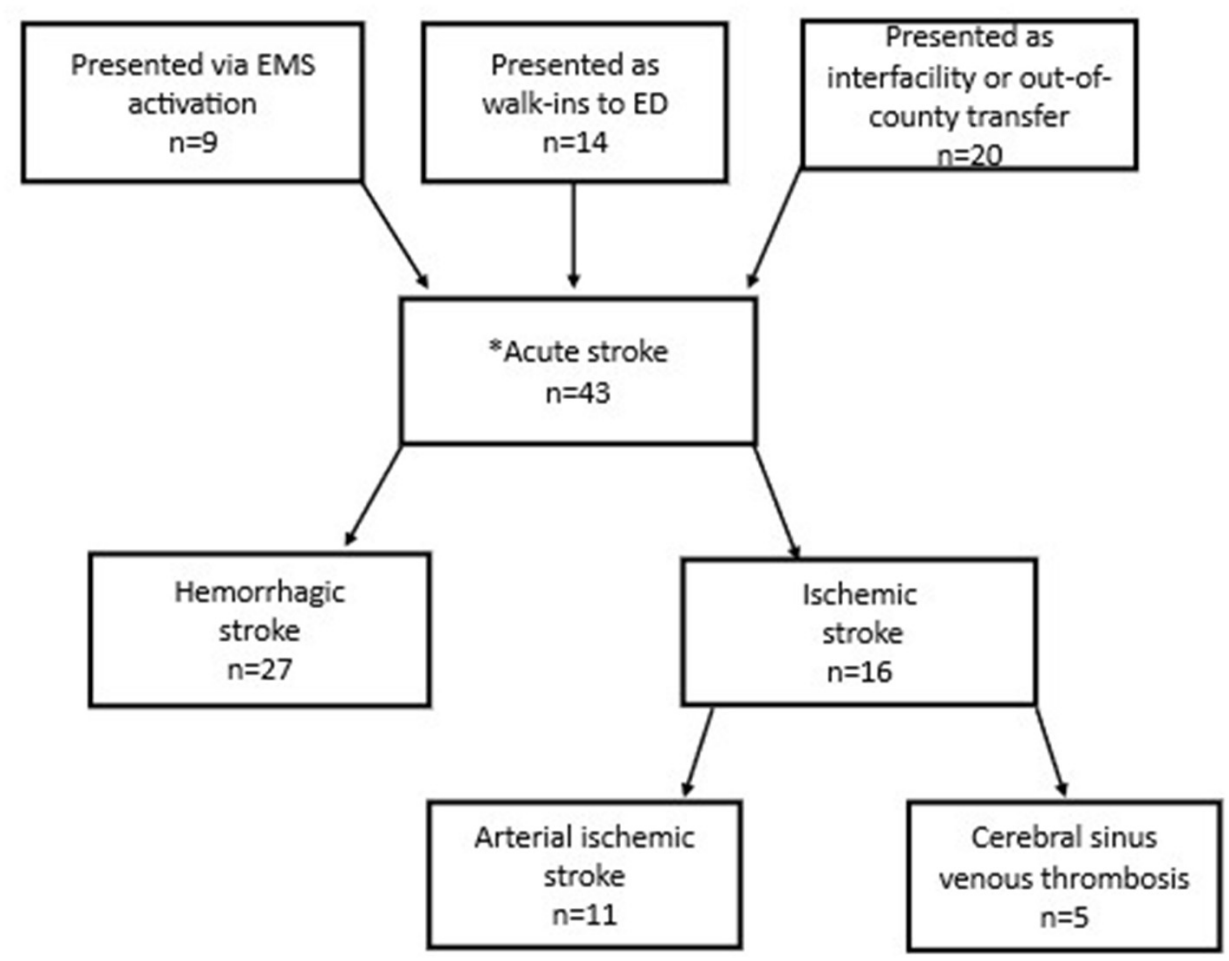
Flow diagram of presentation modality of 43 patients (1–14 years old) with out-of-hospital strokes. *Inpatient strokes excluded.

Our secondary cohort included 43 children with confirmed out-of-hospital acute stroke (Figure 3). Nine (21%) of these were the children that presented via EMS activation and were captured in the primary cohort. An additional 14 (33%) children with acute stroke were identified who were taken to the ED by family/caregivers (“walk-ins”). Twenty (47%) children with stroke were identified who were transferred from another medical center. Overall, 27 cases (63%) had hemorrhagic strokes and 16 (37%) had ischemic strokes (e.g., arterial ischemic stroke, cerebral sinus venous thrombosis). Of those who were walk-ins to the ED, 8 patients (57%) had hemorrhagic stroke, and 6 patients (43%) had ischemic stroke.

All children with out-of-hospital acute stroke who presented via EMS activation had one of four primary symptom or impression codes: (1) seizure; (2) altered mental status; (3) loss of consciousness; (4) headache. For those that presented as walk-ins to the ED, the most common chief complaints were head injury and general neurologic complaint. Times from ED arrival to neuroimaging are shown in Table 4.

**Table 4:** Time from ED arrival to neuroimaging.

**Stroke type**	**Total**			**Arrival by EMS**			**Arrival by walk-in**			***p-value***
	***N***	**Median**	**(IQR)**	***N***	**Median**	**(IQR)**	***N***	**Median**	**(IQR)**	
**Time from ED arrival to CT, in hours**										
Ischemic	5	1.02	(0.82, 1.50)	2	1.16	(0.82, 1.50)	3	1.02	(0.23, 52.1)	1.00
Hemorrhagic	16	1.31	(0.92, 5.73)	7	0.92	(0.47, 1.08)	9	5.69	(1.50, 9.76)	0.002
**Time from ED arrival to MRI, in hours**										
Ischemic	6	4.15	(2.79, 17.6)	2	4.15	(3.00, 5.31)	4	10.2	(1.99, 36.3)	1.00
Hemorrhagic	14	51.1	(10.6, 208)	7	127	(21.4, 577)	7	35.6	(6.6, 76.5)	0.26

## Discussion

In this large retrospective cohort of children with EMS activations for acute neurological symptoms, fewer than one in five underwent neuroimaging. Of those who were imaged, four of five received only CT (and not MRI), despite MRI being the preferred imaging modality for diagnosing pediatric ischemic stroke (Harrar et al., 2022; Mirsky et al., 2017; Christy et al., 2018). While many children with acute neurological symptoms may not warrant imaging (e.g., if symptoms are improving and/or the exam is reassuring), for those who exhibit persistent and concerning neurological symptoms, timely imaging is essential to determine a stroke diagnosis and initiate therapeutic interventions.

EMS providers rarely suspected pediatric stroke: it was the EMS primary impression code in only 13 patients (less than 1% of all EMS activations for acute neurological symptoms in children). Of note, none of these patients were diagnosed with stroke. However, when EMS did suspect stroke, their framing had an impact: all 13 patients received neuroimaging. This suggests that anchoring bias, or relying too heavily on the first piece of information offered, may occur when pediatric stroke is considered as part of the differential diagnosis early in the course of care (Ly et al., 2023). That is to say, when ED providers see this primary impression code documented by pre-hospital providers, they may be more inclined to have stroke on their own differential (or higher on their differential). While we often think of “bias” as inherently negative, in this case, relying on the EMS suspicion of stroke appears to increase clinician awareness of this rare, but high-stakes, possible diagnosis. This suggests an important opportunity: efforts to increase awareness of pediatric stroke and diagnostic accuracy amongst EMS providers could help reduce the time to stroke diagnosis.

Patient age also played a role in who was selected for neuroimaging: children under 5 years old were less likely to undergo neuroimaging than others. Previous studies have shown that younger children are at increased risk of ischemic stroke compared to older children; they also tend to be more difficult to examine, so clinicians may have a lower threshold to image younger patients (deVeber et al., 2017; Oleske et al., 2021; Tan et al., 2022). However, some providers may be reluctant to obtain neuroimaging for younger children due to concerns related to radiation or potential need for sedation to avoid movement artifact. In addition, providers may be less likely to consider hyperacute interventions in younger patients.

Hispanic children were significantly less likely to have neuroimaging compared to children of other races/ethnicities, even after accounting for primary spoken language, although clinically it was a modest difference (79% vs. 85%). While one study on racial and ethnic disparities in childhood stroke found that Hispanic children were relatively protected for all stroke types (relative risk 0.76 compared to White children), this difference should not warrant forgoing imaging in these children where stroke is still a possibility and a “cannot miss” diagnosis (Fullerton et al., 2003). Moreover, in line with other studies that have analyzed final diagnoses in children with acute neurological symptoms (Mackay et al., 2014, 2016), we identified a significant subset of children with actionable abnormal MRIs with findings other than stroke, emphasizing the importance of neuroimaging for select children presenting with acute neurological symptoms. Prior studies have similarly found that Hispanic children and non-Hispanic Black children are less likely to receive diagnostic imaging in the ED compared with non-Hispanic white children (Marin et al., 2021). Further research is needed to better understand the factors that contribute to this trend.

### Primary cohort: predictors of MRI acquisition and abnormal MRI

Factors associated with MRI acquisition were prior history of malignancy and primary impression code of stroke. The latter finding likely reflects existing guidelines that recommend MRI as the preferred imaging modality for pediatric stroke (Mirsky et al., 2017; Khalaf et al., 2018).

Conversely, we found that certain factors were associated with not obtaining an MRI: having an EMS primary symptom code of “focal neurological deficit” or primary impression code of “altered level of consciousness” or “anxiety.” The primary impression code of “anxiety” may lead to diagnostic overshadowing bias, in which clinicians presume that symptoms have a psychological, rather than a structural or neurological etiology, particularly in patients with a pre-existing mental health diagnosis (Molloy et al., 2023). Patients who presented with focal neurological deficits or altered level of consciousness were more likely to have evidence of hemorrhagic stroke on CT, such that MRI was unnecessary for diagnosis (Jordan and Hillis, 2007). Furthermore, for those who had MRI, it was often delayed with a median door-to-imaging time >24 hours from EMS activation. This is consistent with findings from prior studies demonstrating that pediatric ischemic stroke diagnosis is often delayed by approximately 24 hours from symptom onset.

### Secondary cohort: presentation modality and imaging characteristics in children with out-of-hospital acute stroke

To determine whether the imaging practices that we identified were generalizable to all children with out-of-hospital acute stroke, regardless of mode of presentation, we performed a secondary analysis of all children admitted with out-of-hospital stroke. Somewhat surprisingly, <25% of children presented via EMS activation; more children presented either as walk-ins or as transfers to the ED. The substantial proportion of patients who presented as walk-ins (33% of all cases) may reflect the general lack of caregiver awareness that children can have strokes.

Interestingly, most patients with stroke who presented via EMS had hemorrhagic stroke, rather than ischemic stroke, while the distribution between hemorrhagic vs. ischemic stroke was more evenly balanced among walk-ins. This may be because ischemic strokes are often secondary to arteriopathy in children and have a more subtle or “stuttering” presentation compared to hemorrhagic strokes which typically present with lethargy, severe headache with vomiting, and/or altered mental status (Yock-Corrales et al., 2018; Pangprasertkul et al., 2022; Braun et al., 2007; Rafay et al., 2020; Wintermark et al., 2017; Broderick et al., 1993). Hemorrhagic stroke symptoms may be more alarming to caregivers and more likely to precipitate a call to EMS.

In the secondary cohort, patients with stroke who presented via EMS generally had a shorter door to diagnosis time than those who came to the ED as walk-ins. Notably, median time to MRI appeared to be faster for children with ischemic stroke compared to those with hemorrhagic stroke; children with hemorrhagic stroke had MRI done after acute interventions or later in their hospitalization as part of the diagnostic work-up to identify the etiology of hemorrhage and not done as part of the initial diagnosis or to guide hyperacute management. For these children with hemorrhagic stroke, the time to CT is a more meaningful metric than time to MRI for assessing the efficacy of the acute stroke care pathway.

### Limitations

Our study has several limitations. Of note, the overall breakdown of hemorrhagic (62.8%) vs. ischemic (37.2%) stroke in our study was skewed to a higher percentage of hemorrhagic strokes compared to the 50–50 split reported in most pediatric stroke studies (Jordan and Hillis, 2007; Broderick et al., 1993, 1989). While the reason for this is not clear, some factors potentially playing a role are our exclusion of patients over 14 years of age and limitation of our secondary analysis to those with out-of-hospital strokes. Notably, a recent paper by Tan et al. (2022) found a similar distribution of hemorrhagic (69.4%) vs. ischemic (30.5%) stroke in children presenting to the ED.

Due to significant variability in the structure of EMS and pediatric receiving centers across the nation, our findings may not be generalizable to other counties or regions. In addition, we only included patients who were taken to the primary CCERC in Orange County, which is staffed by pediatric specialists and has a pediatric “code stroke” pathway to identify children with possible stroke and thus expedite imaging (see Supplementary material). Findings would likely differ were we to study adult stroke centers and/or community hospitals that primarily serve adult patients. Our study site also has 24/7 MRI capabilities but requires a technician to drive in from offsite during off hours. Differences in imaging capabilities could also limit the generalizability of our findings to other hospital systems. In addition, due to the retrospective nature of the study, we were limited in our ability to establish cause and effect due to missing data. Specifically, the children with out-of-hospital strokes who were transferred from outside facilities often had imaging prior to transfer. Therefore, they were excluded from our analysis of time to imaging at our institution. Our sub-analyses of children who received neuroimaging and of children with stroke were both limited by small sample size.

## Conclusions

Our study highlights some of the differences and challenges in diagnosing acute pediatric stroke and demonstrates opportunities to reduce the time to diagnosis. While EMS providers rarely suspected stroke, their diagnosis impacted imaging decisions in the ED. In other words, when EMS “frames” a child as a potential acute stroke patient, the ED responds accordingly and obtains neuroimaging. Hence, educational programs that raise awareness of pediatric stroke among prehospital providers would likely lead to more rapid diagnosis.

Our study also highlights the need for a unique metric to measure the effectiveness of care pathways for children presenting with acute stroke-like symptoms. We propose that future studies further explore “door to diagnosis” as a meaningful metric for pediatric stroke. This metric accounts for the relevance of CT in diagnosing hemorrhagic stroke vs. MRI in diagnosing ischemic stroke. MRI is considered essential to determine eligibility for hyperacute interventions in the absence of a large vessel occlusion. In cases of hemorrhagic stroke, CT often suffices to establish a diagnosis and acute treatment plan. Healthcare practitioners must acknowledge the complexity of diagnostic imaging for pediatric stroke in order to accurately appraise existing care pathways and determine whether they are successfully achieving the goal of timely stroke diagnosis and early intervention. The door-to-diagnosis metric captures this complexity and could serve as a meaningful endpoint that we can aim to improve upon to optimize clinical outcomes of children with stroke.

## Data Availability

The raw data supporting the conclusions of this article will be made available by the authors, without undue reservation.
