# Supplementary material for: Striving toward quality metrics for pediatric stroke: time from door to diagnosis
Source: Front Stroke. 2026 Jan 7;4:1718355. doi: 10.3389/fstro.2025.1718355 (PMC12802760; doi:10.3389/fstro.2025.1718355)
Supplement: Supplementary file 1 [file Table_1.docx]

Supplementary Materials and Methods

Search Terms Used to Identify Patients with Acute Neurological Symptoms

The dispatch chief complaint is coded by the EMS dispatcher that receives the 911 call. The primary symptom code, primary impression code, and EMS narrative are entered by the EMS clinician upon his or her evaluation of the patient. The ED discharge ICD-10 code is coded by the emergency department attending physician at the end of the ED visit.

| **EMS Data (OC-Meds Label)** | | | | **ED Discharge ICD-10 Codes** |
| --- | --- | --- | --- | --- |
| **Dispatch Chief Complaint** | **Primary Symptom Codes** | **Primary Impression Codes** | **EMS Narrative Terms** |  |
| Stroke/CVA | Altered Mental Status  Aphasia (inability to speak)  Confusion  Difficulty walking  Dizziness  Facial droop  Headache (head pain, medical – non-traumatic)  Hemiplegia  Paralysis of one limb  Seizure  Slurred speech  Visual disturbance  Visual loss  Weakness | Alcohol intoxication  Altered level of consciousness/lethargic (not hypoglycemia or seizure)  Anxiety reaction/emotional upset  Dizziness/vertigo  Drug overdose/ poisoning  Headache  Seizure – active  Seizure – postictal  Stroke (CVA/TIA) | Aphasia  Speech  Slurred  Dysarthria  Dysphagia  Hemiparesis  Hemiplegia  Droop  Vertigo  Stroke | I61 series  I63 series  I67 series  I69 series  G45.9  Q27.3  Q28.3 |

ICD-10 Codes used to identify children admitted with out-of-hospital stroke
